# Differential diagnoses of MS in clinical practice: incidence and methods for differential diagnosis

**DOI:** 10.3389/fneur.2025.1695071

**Published:** 2025-12-04

**Authors:** Tim Johannes Steinberg, Lucie Mayer, David Freudenstein, Karolina Müller, Klemens Angstwurm, Ralf A. Linker, De-Hyung Lee

**Affiliations:** 1 Department of Neurology, University Medical Center Regensburg, Regensburg, Germany; 2 Center for Clinical Studies, University Medical Center Regensburg, Regensburg, Germany

**Keywords:** differential diagnoses, multiple sclerosis, duplex ultrasound, cerebrospinal fluid, cranial MRI, multiple sclerosis mimics

## Abstract

**Background and objectives:**

The diagnosis of multiple sclerosis (MS) is based on the McDonald diagnostic criteria and the exclusion of relevant differential diagnoses. This study aimed to determine the relative frequencies of MS differential diagnoses and to identify which diagnostic measures are most effective in distinguishing those from one another.

**Methods:**

We conducted a retrospective analysis of all cases treated in the neurology ward of the University Hospital Regensburg during the years 2019 and 2020. The inclusion criteria comprised cases presenting with subacute focal neurological symptoms accompanied by corresponding lesions on magnetic resonance imaging (MRI) that could not be primarily attributed to MS, tumor, hemorrhage, or ischemia, as well as cases with abnormal external MRI findings who were referred to our clinic for the evaluation or exclusion of multiple sclerosis.

**Results:**

A total of 127 cases were included in the study. Differential diagnoses of MS were predominantly of inflammatory or vascular origins or non-specific white matter lesions. The primary diagnostic tools for distinguishing differential diagnoses of MS from one another were patient history (fever), cerebrospinal fluid analysis, and brain MRI, as well as a duplex ultrasound of the cranial vessels.

**Discussion:**

Our study demonstrates that cerebrospinal fluid analysis and brain MRI are essential not only for the diagnosis of MS but also for distinguishing among its various differential diagnoses. In particular, duplex ultrasound may assist in identifying vascular lesions, while the presence of fever may suggest an infectious cause.

## Introduction

The diagnosis of multiple sclerosis (MS) is based on the McDonald criteria (1, 2). The authors of the 2024 revision emphasized the importance of ruling out potential differential diagnoses—sometimes referred to as MS mimics—before applying these criteria. A recent review highlighted the broad spectrum of differential diagnoses of MS, which includes other autoimmune disorders, vascular diseases, infections, neoplasms, metabolic conditions, and neurodegenerative diseases (3). The review also provided recommendations regarding hallmarks in clinical presentation, neuroimaging, and cerebrospinal fluid analysis to aid in their differentiation. Further data have been published to help in differentiating MS from its differential diagnoses based on cerebral magnetic resonance imaging (cMRI) (4) and cerebrospinal fluid (CSF) parameters (5, 6). Recently, Solomon et al. (3) published a consensus statement outlining the diagnostic pathway for these conditions (7).

Despite these studies, little is known about the relative frequency of the differential diagnoses of MS when confirming or excluding MS in clinical practice, or about the diagnostic value of various paraclinical tests in distinguishing among them. This study aimed to address this knowledge gap.

## Methods

### Case selection

In this retrospective analysis, all patients treated in the neurology ward of the University Hospital Regensburg between 1 January 2019 and 31 December 2020 were screened. The study aimed to include all patients with clinical and/or radiological signs suggestive of MS who were ultimately diagnosed with conditions other than MS. Case selection was performed by an experienced neurologist (TS). All cases with a first diagnosis of MS, including radiologically isolated syndrome (RIS) and clinically isolated syndrome (CIS), neuromyelitis optica spectrum disorder (NMOSD), and myelin oligodendrocyte glycoprotein antibody-associated disease (MOGAD) were excluded prior to the study. Cases were included if they presented with any subacute (a duration of less than 6 months) focal neurological symptoms and corresponding cerebral magnetic resonance imaging (cMRI) lesions. Additionally, we included all cases referred to our ward due to abnormal cMRI findings for the evaluation or exclusion of an MS diagnosis. When the initial written neuroradiological evaluation of computed tomography (CT) or MRI revealed acute ischemia, hemorrhage, or neoplasm, patients were excluded.

### Case analysis

We analyzed all patient records from both inpatient treatment and outpatient visits to our neurology clinic between 1 January 2019 and 31 December 2020. The records were reviewed for predefined clinical history, neurological symptoms, laboratory findings, cerebrospinal fluid (CSF) analysis, electrophysiology, and imaging data. For statistical analysis, all data were categorized into dichotomous variables (for example, pathological–not pathological). For a comprehensive overview of all evaluated parameters and cutoffs, see Table 1. The final diagnosis was made after evaluation of all clinical and paraclinical data and not solely based on cMRI findings.

**Table 1:** Overview of all anamnestic and diagnostic parameters investigated in all cases.

General diagnostic methods	Specific diagnostic methods
Baseline characteristics	Sex Age
Anamnestic information	Prior diagnosis relevant for final diagnosis Prior medication relevant for final diagnosis Smoking Headache
CSF	WBC (>ULN) Lactate concentration (>ULN) Protein concentration (>ULN) Albumin quotient (>ULN) OCB (type II or III) Autochthonous immunoglobulin synthesis (>ULN) MRZH reaction (at least two pathological values) FACS (showing monoclonal cells) CSF microscopy (showing malignant cells) Evidence of specific infectious disease (PCR or ASI) Protein 14-3-3 (>ULN) Evidence of antineuronal or paraneoplastic antibodies
Blood workup	Beta 2 GP 1 antibodies (>ULN) Lupus anticoagulant (>ULN) Cardiolipin antibodies (>ULN) Complement C1/C3/C4 (<LLN) ANA (>1:160) SSA (>ULN) SSB (>ULN) cANCA (>ULN) pANCA (>ULN) ACE (>ULN) sIL2-R (>ULN) Neopterin (>ULN) Lysozyme (>ULN) ESR (>50 mm/h) Vitamin B12 (<LLN) Folic acid (<LLN) creatinine (> 2ULN) GOT (>2 ULN) GPT (>2ULN) Proteinuria (>ULN) Immunoglobulins against *Borrelia burgdorferi* HIV test *Treponema pallidum* test Specific tests for leukodystrophies
Imaging	cMRI (neuroradiological evaluation consistent with final, clinical diagnosis) Spinal MRI (showing any lesions) Chest CT (showing neoplasia or bilateral hilar lymphadenopathy) Chest and abdominal CT (showing neoplasia) PET CT (showing neoplasia) DSA (indicative of vasculitis)
Others	Presence of fever on admission or shortly prior EP (showing demyelinating damage) Duplex ultrasound of the cranial vessels (showing arteriosclerosis or vascular stenosis) Duplex ultrasound of the temporal artery (indicative of giant cell arteritis) Brain biopsy (indicative of final diagnosis) Pathergy test

For laboratory and imaging data, specific cutoffs are provided. Prior diagnoses were considered relevant to the final diagnosis when chronic conditions were identified as the cause of MRI lesions, including risk factors for vascular lesions (e.g., systemic lupus erythematosus in neuropsychiatric lupus, hypertension in cases with vascular lesions). Previous medication was also deemed relevant if it contributed to the lesions observed on cMRI (e.g., cyclophosphamide-induced white matter lesions). ULN, upper laboratory limit; LLN, lower laboratory limit; WBC, white blood cell count; OCB, oligoclonal bands; MRZH, measles, rubella, varicella zoster, herpes simplex; FACS, fluorescence-activated cell sorting; PCR, polymerase chain reaction; ASI, antibody specificity index (ASI quantifies intrathecal antibody synthesis by comparing the ratio of antibody concentration in CSF to serum against the ratio of total IgG in CSF to serum. This is a relative measure of specific intrathecal antibody production against pathogens and thus an indicator of a recent or past CNS infection); beta 2 GP1, beta 2 glycoprotein 1; ANA, antinuclear antibodies; SSA, anti-Sjögren’s syndrome-related antigen A autoantibodies; SSB, anti-Sjögren’s syndrome-related antigen B autoantibodies; cANCA, antineutrophil cytoplasmic antibodies; pANCA, perinuclear antineutrophil cytoplasmic antibodies; ACE, angiotensin-converting enzyme; sIL2-R, soluble interleukin 2 receptor; ESR, erythrocyte sedimentation rate; GOT, glutamate–oxaloacetate transaminase; GPT, glutamate–pyruvate transaminase; HIV, human immunodeficiency virus; CT, computed tomography; PET-CT, positron emission tomography–computed tomography; DSA, digital subtraction angiography; EP, evoked potentials.

### Statistical analyses

Statistical analyses were conducted using SPSS Statistics 26 (SPSS Inc., Chicago, Illinois). The results are presented as absolute numbers, absolute and relative frequencies (*n*, %), and medians with interquartile ranges (IQR). To analyze the relationship between the categorical variables, the Pearson chi-square test was conducted. Since expected cell frequencies below five occurred in the crosstab, the Monte-Carlo method with 10,000 replications was used to estimate the significance level. In the case of a significant result, an exploratory *post-hoc* analysis at the cell level was performed, based on the standardized residuals. Cells with a residual greater than |1.96| were interpreted as significantly deviating from the expected value. This was an exploratory data analysis without adjusting for multiple testing.

### Ethical approval

The Ethics Committee at the University of Regensburg approved the study (#24-3,839-104). The analysis was conducted in accordance with the current Declaration of Helsinki.

## Results

### Cohort description

**Figure 1:**
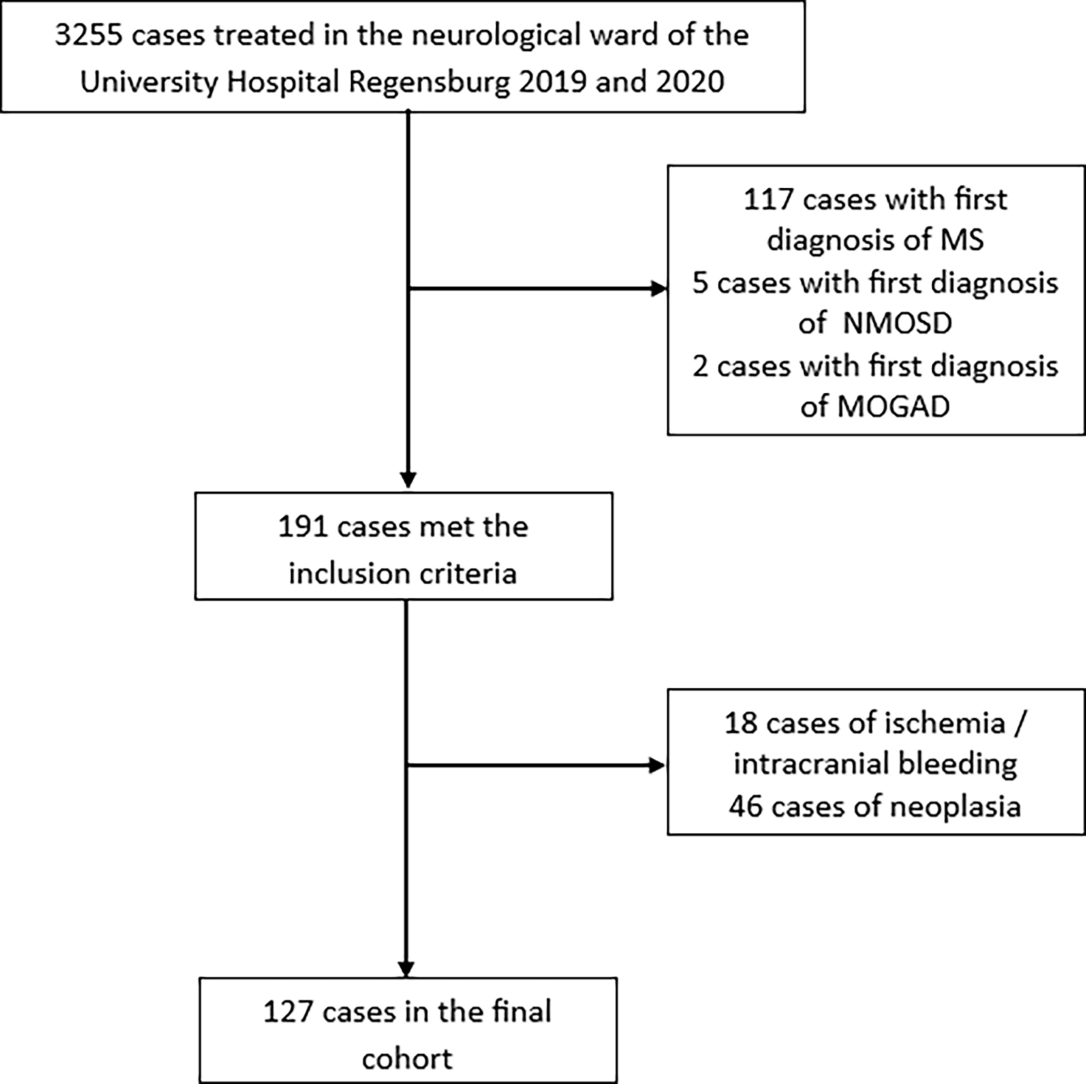
Flowchart regarding inclusion of patients. Excluded cases labeled as MS also comprised individuals with radiologically isolated syndrome (RIS) and clinically isolated syndrome (CIS). Cases with ischemia, intracranial bleeding, or neoplasia were excluded only if the initial imaging (CT or MRI) clearly indicated these conditions.

A total of 3,255 cases were screened, of which 127 (3.9%) met the predefined criteria (see Figure 1). Of those, 61% were women, and the mean age was 50.9 years with a standard deviation of 18.2 years. In 53% of the cases (*n* = 67), patients were referred to our clinic due to white matter lesions of unknown origin identified on recent cMRI, typically for the evaluation or exclusion of MS. In 30% of the cases (*n* = 38), patients presented independently or were referred because of subacute neurological deficits, which were later attributed to specific lesions on cMRI. Both criteria were met in 22 cases (17%).

In our cohort, 12 cases (9%) had autoimmune diseases, and 20 cases (16%) had infectious diseases. Among the 12 cases with autoimmune conditions, the following diagnoses were identified: autoimmune encephalitis (five cases), systemic lupus erythematosus (two cases), Rasmussen’s encephalitis, microscopic polyangiitis, Bickerstaff encephalitis, sarcoidosis, and Behçet’s disease. Infectious etiologies included John Cunningham (JC) virus (three cases), Creutzfeldt–Jakob disease (CJD, five cases), cerebral abscesses (three cases), herpes simplex virus (HSV) type 1 (three cases), *Borrelia burgdorferi*, tick-borne encephalitis, *Capnocytophaga canimorsus*, and three cases in which no specific microorganism could be identified, although infectious encephalitis was strongly suspected. Vascular lesions were identified in 27 cases (22%); 14 patients were diagnosed with postischemic lesions, 11 had microangiopathic white matter lesions, and 1 case each was diagnosed as giant cell arteritis and primary angiitis of the central nervous system (PACNS).

Non-specific white matter lesions were observed in 19 cases (15%). In 32 cases (25%), no diagnosis could be made, and therefore, they remained unsolved.

In the remaining cases, the following diagnoses were made: gliomatosis cerebri, hemangioblastoma, neoplasm of unknown origin, central pontine myelinolysis (four cases), cytotoxic edema due to hyperthermia, telangiectasia, cavernoma, migration abnormality, cyclosporine-induced neurotoxicity, posterior reversible encephalopathy syndrome (PRES), and post-traumatic lesions. In three cases, previously reported external cMRI lesions could not be confirmed upon re-evaluation. Notably, in three cases diagnosed with neoplasia, diagnoses could only be established later in the disease course and were not apparent on the initial cMRI scan.

### Diagnostic groups

Initial analysis showed a broad variety of differential diagnoses. For further statistical analysis, subgroups had to be defined. Based on the most frequent diagnoses, we defined six diagnostic groups: autoimmune, infectious, vascular, non-specific lesions, others, and unsolved cases. The absolute and relative frequencies within these groups are presented in Table 2.

**Table 2:** Absolute and relative frequencies of diagnoses in our cohort.

Diagnostic group	Absolute frequency	Relative frequency in %
Autoimmune	12	9.4
Infectious	20	15.7
Vascular	27	21.3
Non-specific white matter lesions	19	15.0
Other diagnoses	17	13.4
Unsolved	32	25.2

All 127 cases were assigned to one of the six diagnostic groups. The absolute number of cases in each group, along with their relative frequencies within the entire cohort, is reported.

### Diagnostic evaluation

In the second step, we aimed to examine which diagnostic measures were most reliable to distinguish differential diagnoses of MS from one another. The Pearson chi-squared test with the Monte Carlo method was used for statistical analysis.

#### History

Prior medical conditions were most relevant in the context of autoimmune diseases and vascular diseases (*p* < 0.001). In vascular diseases, 59% of cases had a history of diabetes mellitus or hypertension. Smoking status and the presence of headache at presentation did not differ significantly across the four groups. However, fever was observed significantly more frequently in cases with infectious diseases of the central nervous system (CNS, *p* = 0.002).

#### CSF

Regarding CSF findings, cases with autoimmune and infectious diseases showed significant differences from the other groups in the following pathological parameters: white blood cell count (WBC) (*p* < 0.001), protein concentration (*p* < 0.001), albumin quotient (*p* < 0.001), lactate concentration (*p* < 0.001), oligoclonal bands (OCBs) (*p* < 0.001), and autochthonous immunoglobulin synthesis (*p* < 0.001). Autochthonous immunoglobulin synthesis is a relative measure of non-specific intrathecal antibody production and thus serves as an indicator of certain autoimmune diseases of the CNS. No significant differences were observed for measles, rubella, varicella zoster, herpes simplex (MRZH) reaction, antineuronal or paraneoplastic antibodies, microscopy, fluorescence-activated cell sorting (FACS), or microbiological evidence of infectious disease. These tests, however, were conducted in only a minority of cases (<30%).

#### Blood tests

Glutamate–pyruvate transaminase (GPT) (*p* = 0.008) elevation was significantly more common in the autoimmune group, whereas proteinuria was more common (*p* = 0.019) in the infectious group. No other blood test results showed significant differences across the four diagnostic groups, especially considering that these tests were performed in only a subset of cases and mostly yielded negative results. Nonetheless, in individual cases, these tests made a significant contribution to distinguishing specific diagnoses within the diagnostic groups.

#### Imaging

MRI played a central role in differentiating between diagnostic groups and in identifying specific diagnoses within each group. In nearly all cases—except those in the group with unsolved diagnoses—neuroradiological diagnosis in cMRI was consistent with the final clinical diagnosis (*p* < 0.001). Duplex ultrasound yielded significantly more pathological results in the group of vascular diseases (*p* = 0.006). Other imaging modalities were used less frequently and did not show significant differences across the diagnostic groups.

For more detailed information about the absolute frequencies, relative frequencies, and *p*-values for each diagnostic modality in every group, please refer to Supplementary Table A1.

## Discussion

This study highlights the heterogeneity of differential diagnoses of multiple sclerosis (MS) in clinical practice. Regarding our cohort, it is important to note that the mean age was relatively high (50.7 years) compared to the average age at the first MS diagnosis (8). This may indicate a significant proportion of atypical cases within our cohort and is reflected in a notably higher frequency of vascular lesions. As MS is increasingly diagnosed in older patients, particularly those aged over 50, the findings of this study become more relevant (8–10). A variety of—at times quite rare—differential diagnoses of MS exist, as highlighted in recent literature (3, 6, 11). When categorized into diagnostic groups, differential diagnoses of MS predominantly arise from inflammatory (autoimmune and infectious) and vascular diseases. However, in a considerable number of cases, no clear underlying condition could be identified, or the lesions were described as non-specific. Such non-specific white matter lesions may occur in disorders such as migraine (12). Surprisingly, the cohort includes five cases of CJD, accounting for 0.2% of all cases treated at our clinic in 2019 and 2020. The current incidence of CJD in Germany is estimated at one to two cases per million inhabitants per year (13). All affected individuals were initially referred to our clinic for the evaluation of focal neurological deficits and were later found to have corresponding lesions on MRI. The disproportionately high frequency of CJD observed in our clinic remains unexplained, though it may reflect a broader underdiagnosis of the condition (14).

One major group in our study comprised unsolved cases. The majority of these patients were referred to our clinic for the assessment of a potential MS diagnosis due to the presence of cMRI lesions. In the majority of cases, no corresponding objective symptoms were identified. As a result, the diagnostic process primarily focused on excluding MS and other treatable causes of the lesions.

Regarding the diagnostics that help to differentiate MS from its differential diagnoses, it is known from the McDonald criteria that MRI and CSF play the most important role here (1, 2). In contrast, it is unknown which diagnostic instruments are helpful to differentiate the heterogeneous group of differential diagnoses of MS from one another. In our analysis, CSF was especially helpful for differentiating inflammatory (both autoimmune and infectious) diagnoses from others. In addition to the expected role of WBC in CSF, elevated protein concentrations, lactate concentrations, and the presence of OCBs bands were also more common in the inflammatory group. The latter finding highlights the importance of considering differential diagnoses of MS, since OCBs, although often regarded as typical for MS, may also occur in other conditions (5).

The second most important diagnostic tool was cMRI, which contributed to establishing the diagnosis in the majority of cases. However, it is important to consider the potential for circular reasoning, as considerable reliance may be placed on the seemingly objective cMRI findings—particularly given the absence of a definitive gold standard for confirming the final diagnosis in our study. The final diagnoses in our study were based on all available clinical data. In some cases, these findings did not align with the neuroradiological diagnosis; however, as radiological findings in cMRI are part of the inclusion criteria and neuroradiological diagnosis in the cMRI part of the results, this potential bias could not be excluded. Unfortunately, our study included only a small proportion of patients who underwent additional spinal MRI. This could serve as a valuable tool for distinguishing differential diagnoses of MS, but further investigation through a dedicated study would be necessary.

Interestingly, duplex ultrasound also proved significantly useful in distinguishing cases with vascular lesions from those with non-vascular findings. Notably, the assessment did not depend on directly related pathologies such as downstream vessel stenosis of MRI pathologies; instead, it focused solely on the presence of arteriosclerosis and any form of vascular stenosis.

Fever was significantly associated with infectious diseases. Prior diagnoses were significantly important regarding cardiovascular risk factors for vascular diseases and prior autoimmune diseases in the group of autoimmune diseases of the CNS.

The liver enzyme GPT was significantly more frequently elevated in cases with autoimmune diseases. The underlying cause of this finding remains unclear, though a potential association with immunosuppressive therapy as well as the involvement of the liver in systemic autoimmune disorders could be suspected. Proteinuria was also significantly more common in the infectious group, although its etiology remains undetermined. We do not believe that these laboratory tests significantly aid in the evaluation of differential diagnoses of MS in clinical practice.

In conclusion, we recommend performing cMRI and CSF analysis not only to differentiate between MS and its mimics but also to distinguish among the various differential diagnoses of MS. Additionally, duplex ultrasound could be considered if vascular origin of lesions is suspected and can, in these cases, support the diagnosis. The occurrence of fever should lead to further tests for infectious causes. However, the presence of fever neither proves an underlying infectious cause nor does its absence rule it out. Other diagnostic procedures, including laboratory workups, should be reserved for selected cases based on clinical suspicion of specific conditions and not be performed as a standard “MS workup.”

Our study is limited by its single-center design and the moderate sample size of 127 cases, particularly in light of the extensive range of rare differential diagnoses. Additionally, due to the lack of a well-defined entity of differential diagnoses of MS, we used broad inclusion criteria to capture a large and heterogeneous cohort. This heterogeneity may have impeded a more precise analysis. Several tests were performed only in selected cases, which likely contributed to the lack of significant findings, particularly regarding laboratory results. Regarding statistical analysis, no adjustment for multiple testing was applied, which increases the risk of Type I errors and implies that some significant results may have occurred by chance. This limitation applies both to the performance of several global tests within the same dataset and to the exploratory *post-hoc* inspection of standardized residuals, which inherently involves multiple comparisons. Accordingly, the reported test results should be viewed as exploratory signals of potential associations under sparse data conditions.

Overall, our study demonstrates that CSF analysis and neuroradiological evaluation of cMRI are crucial for distinguishing MS from its differential diagnoses, as well as differentiating between these conditions. Clinicians should also consider the potential value of fever as an anamnestic clue and the use of duplex ultrasound when vascular lesions are suspected.

## Data Availability

The original contributions presented in the study are included in the article/Supplementary material, further inquiries can be directed to the corresponding authors.

## Glossary

ACE: angiotensin-converting enzyme
ANA: antinuclear antibodies
ASI: antibody specificity index
Beta 2 GP1: beta 2 glycoprotein 1
CJD: Creutzfeldt–Jakob disease
CNS: central nervous system
CSF: cerebrospinal fluid
CT: computed tomography
DSA: digital subtraction angiography
EP: evoked potentials
ESR: erythrocyte sedimentation rate
FACS: fluorescence-activated cell sorting
GOT: glutamate–oxaloacetate transaminase
GPT: glutamate–pyruvate transaminase
HIV: human immunodeficiency virus
IQR: interquartile ranges
JC virus: John Cunningham virus
LLN: lower laboratory limit
MOGAD: myelin oligodendrocyte glycoprotein antibody-associated disease
MRI: magnetic resonance imaging
MRZH: measles, rubella, varicella zoster, herpes simplex
MS: multiple sclerosis
NMOSD: neuromyelitis optica spectrum disorder
OCB: oligoclonal bands
PACNS: primary angiitis of the central nervous system
pANCA: perinuclear antineutrophil cytoplasmic antibodies
PCR: polymerase chain reaction
PET-CT: positron emission tomography–computed tomography
PRES: posterior reversible encephalopathy syndrome
sIL2-R: soluble interleukin 2 receptor
SSA: anti-Sjögren’s syndrome-related antigen A autoantibodies
SSB: anti-Sjögren’s syndrome-related antigen B autoantibodies
ULN: upper laboratory limit
WBC: white blood cell count
