# Supplementary material for: Differential diagnoses of MS in clinical practice: incidence and methods for differential diagnosis
Source: Front Neurol. 2025 Dec 4;16:1695071. doi: 10.3389/fneur.2025.1695071 (PMC12711508; doi:10.3389/fneur.2025.1695071)
Supplement: Supplementary file 1 [file Table_1.pdf]

Supplementary Table 1: The relative frequency of pathological findings within each diagnostic group (autoimmune, vascular, etc.), along with the significance level based on the Chi-Square test (Monte Carlo estimation, 10,000 replications, exploratory post-hoc inspection of standardized residuals, exploratory analyses, no adjustment for multiple testing) is reported. If no *p*-value is shown, the Chi-Square test was not applicable. If Chi-Square test showed p < 0,05, post-hoc analysis was performed and groups significantly differing from the others are marked with *. The proportion of cases in which the examination was performed is indicated in parentheses for each group.

Diagnostic	autoimmune<br>n = 12	infectious<br>n = 20	vascular<br>n = 27	unspecific<br>n = 19	unsolved<br>n = 32	others<br>n = 17	p-value
**History**							
Prior diagnosis	50% (100%)*	20% (100%)	59% (100%)*	5% (100%)*	0% (100%)*	18% (100%)	<0.001
Prior medication	0% (100%)	15% (100%)*	0% (100%)	0% (100%)	0% (100%)	6% (100%)	0.024
Nicotin	27% (92%)	36% (70%)	41% (81%)	25% (84%)	27% (81%)	33% (71%)	0.892
Headache	86% (58%)	89% (45%)	75% (30%)	89% (47%)	87% (47%)	100% (47%)	0.811
Fever	22% (75%)	57% (70%)*	12% (63%)	0% (42%)	0% (47%)*	25% (24%)	0.002
**CSF**							
CSF WBC	75% (100%)*	53% (95%)*	4% (89%)*	6% (95%)	19% (97%)	6% (94%)	<0.001
CSF protein	67% (100%)*	63% (95%)*	25% (89%)	6% (95%)*	45% (97%)	25% (94%)	<0.001
CSF QAlb	67% (100%)*	58% (95%)*	21% (89%)	6% (95%)*	39% (97%)	13% (94%)	<0.001
CSF lactate	58% (100%)*	32% (95%)*	8% (89%)	0% (95%)	3% (97%)	0% (94%)	<0.001
CSF OCB	50% (100%)*	32% (95%)*	4% (89%)	0% (95%)	6% (100%)	6% (94%)	<0.001
CSF autochthone immunoglobulins	42% (100%)*	32% (95%)*	4% (89%)	6% (95%)	3% (97%)	6% (94%)	<0.001
CSF MRZH	50% (17%)	40% (25%)	0% (0%)	0% (5%)	0% (13%)	0% (12%)	0.492
CSF FACS	0% (33%)	0% (10%)	0% (4%)	0% (0%)	0% (6%)	0% (6%)	-
CSF Microscopy	0% (50%)	0% (10%)	0% (19%)	0% (0%)	0% (22%)	0% (29%)	-
CSF PCR /ASI	14% (58%)	64% (70%)	0% (4%)	0% (5%)	22% (9%)	33% (18%)	0.124
CSF protein 14-3-3	100% (8%)	100% (25%)	0% (4%)	0% (0%)	0% (0%)	0% (6%)	0.292
CSF antibodies	20% (83%)	20% (25%)	50% (7%)	0% (11%)	13% (25%)	24% (24%)	0.964
**Blood tests**							
Beta2-GP1	0% (50%)	0% (15%)	0% (37%)	0% (89%)	5% (63%)	0% (24%)	1.000
Lupusanticoagulant	20% (42%)	0% (10%)	0% (33%)	0% (89%)	0% (59%)	0% (18%)	0.184
Cardiolipin antibodies	0% (0%)	0% (0%)	0% (4%)	0% (5%)	0% (0%)	0% (6%)	-
Complement C1/C3/C5	33% (25%)	100% (10%)	50% (15%)	100% (5%)	50% (13%)	100% (6%)	0.785
ANA	17% (50%)	33% (15%)	0% (48%)	0% (84%)	4% (75%)	0% (29%)	0.105

SSA	0% (17%)	0% (0%)	0% (4%)	0% (26%)	0% (13%)	0% (6%)	-
SSB	0% (17%)	0% (0%)	0% (4%)	0% (26%)	0% (13%)	0% (6%)	-
cANCA	0% (25%)	0% (10%)	0% (30%)	0% (16%)	0% (19%)	0% (12%)	-
pANCA	33% (25%)	0% (10%)	0% (30%)	0% (16%)	0% (19%)	0% (12%)	0.418
ACE	0% (50%)	0% (5%)	0% (37%)	0% (89%)	0% (75%)	0% (24%)	-
sIL2-R	0% (25%)	100% (4%)	0% (4%)	0% (0%)	25% (13%)	0% (0%)	0.502
Neopterin	50% (17%)	0% (0%)	0% (0%)	0% (0%)	50% (13%)	0% (0%)	1.000
Lysozyme	0% (17%)	0% (0%)	0% (0%)	0% (0%)	33% (9%)	0% (0%)	1.000
ESR	0% (0%)	0% (0%)	100% (4%)	0% (5%)	50% (6%)	0% (6%)	1.000
Vitamin B12	0% (17%)	0% (20%)	0% (52%)	6% (89%)	4% (75%)	0% (35%)	1.000
Folic Acid	100% (8%)	0% (15%)	20% (19%)	0% (5%)	0% (6%)	0% (0%)	0.380
Borrelia immunoglobulins	0% (33%)	33% (15%)	13% (30%)	27% (58%)	8% (38%)	33% (18%)	0.631
HIV test	0% (25%)	0% (40%)	0% (4%)	0% (5%)	0% (6%)	0% (6%)	-
Treponema pallidum test	0% (25%)	0% (5%)	0% (4%)	0% (11%)	0% (13%)	0% (6%)	-
Creatinine	8% (100%)	0% (100%)	4% (100%)	0% (100%)	3% (100%)	0% (94%)	0.688
GOT	17% (100%)	5% (100%)	4% (100%)	0% (100%)	0% (100%)	0% (94%)	0.068
GPT	33% (100%)*	10% (100%)	4% (100%)	0% (100%)	3% (100%)	6% (94%)	0.008
Proteinuria	28,6% (58%)	63% (40%)*	20% (37%)	0% (63%)*	20% (63%)	0% (39%)	0.019
Leucodystropy test	0% (0%)	0% (0%)	0% (0%)	0% (0%)	100% (3%)	0% (0%)	-
**Imaging and Electrophysiology**							
cMRI	92% (100%)	95% (100%)*	100% (100%)*	100% (100%)*	0% (100%)*	100% (100%)*	<0.001
sMRI	0% (33%)	0% (0%)	0% (4%)	0% (11%)	8% (41%)	0% (17%)	1.000
CT Thorax	17% (50%)	0% (45%)	25% (15%)	0% (0%)	33% (19%)	0% (35%)	0.329
CT Thorax Abdomen	25% (33%)	40% (25%)	0% (7%)	0% (0%)	50% (13%)	0% (24%)	0.508
PET CT	0% (8%)	0% (0%)	0% (0%)	0% (0%)	0% (13%)	0% (6%)	-
DSA	0% (0%)	0% (0%)	0% (4%)	0% (0%)	0% (3%)	0% (6%)	-
Evoked potentials	67% (25%)	0% (5%)	39% (48%)	6% (84%)	39% (72%)	29% (41%)	0.133
Duplex	100% (17%)	33% (30%)	91% (85%)*	40% (26%)	50% (31%)	33% (17%)	0.006
Duplex temporal artery	0% (0%)	0% (0%)	0% (4%)	0% (5%)	0% (0%)	0% (6%)	-
**Other tests**							
Biopsy	100% (17%)	0% (0%)	100% (4%)	0% (0%)	0% (13%)*	0% (0%)	0.029
Pathergy test	0% (8%)	0% (0%)	0% (0%)	0% (0%)	0% (0%)	0% (0%)	-
